# Antioxidant Potential of *Momordica Charantia* in Ammonium Chloride-Induced Hyperammonemic Rats

**DOI:** 10.1093/ecam/nep227

**Published:** 2011-06-08

**Authors:** A. Justin Thenmozhi, P. Subramanian

**Affiliations:** Department of Biochemistry and Biotechnology, Faculty of Science, Annamalai University, Annamalainagar 608002, Tamil Nadu, India

## Abstract

The present study was aimed to investigate the antioxidant potential of *Momordica charantia* fruit extract (MCE) in ammonium chloride-induced (AC) hyperammonemic rats. Experimental hyperammonemia was induced in adult male Wistar rats (180–200 g) by intraperitoneal injections of ammonium chloride (100 mg kg^−1^ body weight) thrice a week. The effect of oral administration (thrice a week for 8 consecutive weeks) of MCE (300 mg kg^−1^ body weight) on blood ammonia, plasma urea, serum liver marker enzymes and oxidative stress biomarkers in normal and experimental animals was analyzed. Hyperammonemic rats showed a significant increase in the activities of thiobarbituric acid reactive substances, hydroperoxides and liver markers (alanine transaminase, aspartate transaminase and alkaline phosphatase), and the levels of glutathione peroxidase, superoxide dismutase, catalase and reduced glutathione were decreased in the liver and brain tissues. Treatment with MCE normalized the above-mentioned changes in hyperammonemic rats by reversing the oxidant-antioxidant imbalance during AC-induced hyperammonemia, and offered protection against hyperammonemia. Our results indicate that MCE exerting the antioxidant potentials and maintaining the cellular integrity of the liver tissue could offer protection against AC-induced hyperammonemia. However, the exact underlying mechanism is yet to be investigated, and examination of the efficacy of the active constituents of the *M. charantia* on hyperammonemia is desirable.

## 1. Introduction

In mammals, ammonia is an important nitrogen substrate in several reactions and plays an important role in nitrogen homeostasis of mammalian cells. Ammonia is produced by amino acid and protein catabolism and is toxic to brain and muscles. Ammonia toxicity results in free-radical generation that leads to oxidative stress and tissue damage [1]; it is converted to urea in the liver by urea-cycle enzymes, which is then excreted by the kidneys. Hyperammonemia may result from genetic defect or deficiency of the urea-cycle enzymes or from acquired conditions such as Reye's syndrome, liver failure, high-dose chemotherapy and severe infection [2]. Hyperammonemia is a major contributing factor to neurological abnormalities observed in hepatic encephalopathy and congenital defects of ammonia detoxification. Ammonia affects both excitatory and inhibitory synaptic transmission in the mammalian brain through a variety of mechanisms [3]. The clinical features of hyperammonemia are usually nonspecific in adults and include vomiting, lethargy, sleep and behavioral disturbances, hallucinations, delusions and psychosis [4], coma and death [2].

The greatest disadvantage of the currently available potent conventional or synthetic antihyperammonemic agents/therapies lies in their toxicity and reappearance of symptoms after discontinuation. Valporic acid, phenobarbitol and carbamazepine are some of the currently used antiseizure and antihyperammonemic drugs. These drugs or therapies are sometimes inadequate and can have serious adverse effects [5]. Therefore, the screening and development of drugs for their antihyperammonemic activity is still in progress, and there is a growing need to find appropriate protective agents against hyperammonemia from traditional medicinal plants.

*Momordica charantia* L (Bitter melon) is one of the most important species belonging to the family Cucurbitaceae, commonly known as bitter gourd or bitter melon in English. The origin of this crop is presumed to be India, with secondary center of diversity in China [6]. Its fruits, leaves and roots have been shown to exhibit various biological activities, including antidiabetic, antirheumatic, antiulcer, anti-inflammatory and antitumor, and is used for treating jaundice, leprosy and as an antivenom to snakebite [7–9]. A bitter melon fruit has a particular clinical usefulness, similar to MAP30 (*Momordica* anti-HIV protein; molecular weight: 30 kDa) that is believed to have multiple functions that could be beneficial for the treatment of HIV infections [10]. Recently, it has been found to be a powerful activator of peroxisome proliferator-activated *α*-receptor that regulates the expression of genes involved in lipid metabolism and transport [11]. In addition, examination of the phytochemicals of this plant indicated the presence of active components like momorcharins, momordenol, momordicilin, momordicins, momordicinin, momordin, momordolol, charantin, charine, cryptoxanthin, cucurbitins, cucurbitacins, cucurbitanes, cycloartenols, diosgenin, elaeostearic acids, erythrodiol, galacturonic acids, gentisic acid, goyaglycosides, goyasaponins and multiflorenol, which have been isolated [12, 13]. It is a well-documented fact that most medicinal plants are enriched with phenolic compounds and bioflavonoids that have excellent antioxidant property [14, 15].

Although various phytochemical constituents and diverse medicinal activities have been attributed to this plant, no biochemical studies have been carried out to shed light on the role of *M. charantia* fruit extract on liver marker enzymes, lipid peroxidation and antioxidant status in experimental hyperammonemia.

## 2. Methods

### 2.1. Plant Material

The mature green *M. charantia* were collected from Chidambaram, Cuddalore District, Tamil Nadu, India. The plant was identified and authenticated at the Herbarium of Botany Directorate in Annamalai University. A voucher specimen (No. 1260) was deposited at the Botany Department of Annamalai University.

### 2.2. Preparation of Alcoholic Extract (MCE)

Alcoholic extract of the fruit was prepared according to the method developed by Shibib et al. [16]. One kilogram of unripe fruit bought from the local market was thoroughly washed, and the seeds were removed. The pulp was blended in 1500 mL of 95% alcohol, and left at room temperature with occasional shaking for 48 h .The suspension was filtered through cheesecloth, and the filtrate was evaporated in a Rotovac (Buchi Labortechnik AG, Switzerland) at 40°C to remove the alcohol. The final residue was stored at −20°C until further use. The residual extract was suspended in 1% (w/v) carboxymethyl cellulose (CMC), and used in the investigation [17].

### 2.3. Chemicals

Ammonium chloride was purchased from Sisco Research Laboratories, Mumbai, India. All the other chemicals used in the study were of analytical grade.

### 2.4. Animals

Adult male albino Wistar rats, weighing 180–200 g bred in the Central Animal House, Rajah Muthiah Medical College, Annamalai University, were used for the experiment. The animals were housed in polycarbonate cages in a room with a 12-h day-night cycle, at a temperature of 22 ± 2°C and humidity of 45–64%. The animals were fed with a standard pellet diet (Hindustan Lever Ltd, Mumbai, India) and water *ad libitum*. All the animal experiments were approved by the ethical committee, Annamalai University (Clearance No. 536/20-03-08), and were in accordance with the guidelines of the National Institute of Nutrition (NIN), Indian Council of Medical Research (ICMR), Hyderabad, India. Hyperammonemia was induced in Wistar rats by intraperitoneal injections of ammonium chloride at a dose of 100 mg kg^−1^ body weight, thrice in a week for 8 consecutive weeks [18].

### 2.5. Experimental Design

In the experiment, a total of 32 rats were used. The rats were divided into four groups of eight rats each. Group I rats received 1% (w/v) CMC and were considered as control, Group II normal rats were administered with MCE (300 mg kg^−1^ body weight) using an intragastric tube [16], Group III rats were treated with ammonium chloride intraperitoneally (100 mg kg^−1^ body weight) [18] and Group IV rats were treated with ammonium chloride (100 mg kg^−1^) + MCE (300 mg kg^−1^).

At the end of eighth week, the rats were fasted overnight and killed by cervical dislocation after anesthetizing with ketamine hydrochloride (30 mg kg^−1^ body weight; im). Blood was collected, and plasma and serum were separated by centrifugation. The liver and brain tissues were excised immediately and rinsed in ice-chilled normal saline. About 500 mg of the tissues were homogenized in 5.0 mL of 0.1 M Tris-HCl buffer (pH 7.4). The homogenate was centrifuged and the supernatant was used for the estimation of various biochemical parameters.

### 2.6. Biochemical Estimations

Blood ammonia was determined by the enzymatic kinetic colorimetric assay developed by Wolheim [19], using an automated Roche/Hitachi 912 kit. Plasma urea was determined by the diacetyl monoxime method [20] using automated Roche/Hitachi 912 kit. Urea reacted with diacetyl monoxime under strong acidic conditions and produced a pink-colored complex, and the color developed was read at 540 nm. Activities of aspartate transaminase (AST) and alanine transaminase (ALT) were assayed using the method described by Reitman and Frankel [21], while alkaline phosphatase (ALP) was assayed by the method described by King and Armstrong [22].

Plasma thiobarbituric acid reactive substances (TBARS) were estimated by the method developed by Yagi [23], while the TBARS in the liver and brain were estimated by the method developed by Fraga et al. [24]. The estimation of lipid hydroperoxides (HP) in plasma, liver and brain tissues was carried out using the method described by Jiang et al. [25], while the activity of superoxide dismutase (SOD) in the liver and brain tissues was assayed by the method developed by Kakkar et al. [26]. The activity of catalase (CAT) in the liver and brain tissues was assayed by the method described by Sinha [27], and an estimation of reduced glutathione (GSH) in the plasma, liver and brain tissues was made using the method described by Ellman [28]. Furthermore, glutathione peroxidase (GPx) activity was assayed by the method developed by Rotruck et al. [29], and the protein in the enzyme extract was determined by the method described by Lowry et al. [30].

### 2.7. Statistical Analysis

Statistical analysis was performed using one-way analysis of variance (ANOVA), followed by Duncan's multiple range test (DMRT) using SPSS software package 13.0. The results were expressed as mean ± SD from eight rats in each group, and *P*-values of <.05 were considered as significant.

## 3. Results

### 3.1. Antihyperammonemic and Hepatoprotective Effect

Table 1 shows the levels of blood ammonia, plasma urea and serum AST, ALT and ALP of normal and experimental rats. Ammonium chloride-induced (AC) rats showed a significantly increased level of the above-mentioned biochemical parameters when compared with the normal rats. Oral treatment with MCE in AC rats significantly decreased the levels of blood ammonia, plasma urea and serum AST, ALT and ALP, when compared with rats induced with ammonium chloride alone. 

### 3.2. Antilipidperoxidation Activity

Table 2 shows the levels of TBARS and HP in plasma and tissues (liver and brain) of the normal and experimental rats. AC rats showed a significantly increased level of TBARS and HP in plasma and tissues when compared with the normal rats. Oral treatment with MCE in AC rats significantly decreased the levels of TBARS and HP in plasma and tissues (liver and brain) when compared with rats induced with ammonium chloride alone. 

### 3.3. Improvement in Antioxidant Levels in MCE-Treated Rats

The activities of SOD, CAT, GSH and GPx in the liver and brain of the normal and experimental rats are shown in Tables 3 and 4. AC rats exhibited significantly decreased activities of these antioxidant enzymes in the liver and brain when compared with the normal CMC-treated control rats. Furthermore, treatment with MCE in AC rats significantly increased these antioxidants when compared with rats induced with ammonium chloride alone. 

## 4. Discussion

Ammonia is present in all living organisms as a product of degradation of proteins and other nitrogenous compounds. At higher levels, ammonia is toxic, leading to functional disturbances in the central nervous system that could lead to coma and death. To avoid the deleterious effects of ammonia, ureotelic animals detoxify ammonia by incorporating it into urea that is eliminated in urine [31, 32]. Increased levels of circulatory ammonia and urea indicate hyperammonemic condition in AC rats [33], which may be due to liver damage caused by ammonia intoxication.

Numerous investigations have documented that plant extracts containing phenolic compounds and flavonoids offer ammonia detoxification by removing excess ammonia, urea, uric acid and creatinine during various disease conditions, such as hyperammonemia, nephrotoxicity, and so forth [34, 35]. It was reported that MCE could normalize the levels of urea during diabetic conditions in rats [36] and the results of our experiments corroborate these findings.

**Figure 1:**
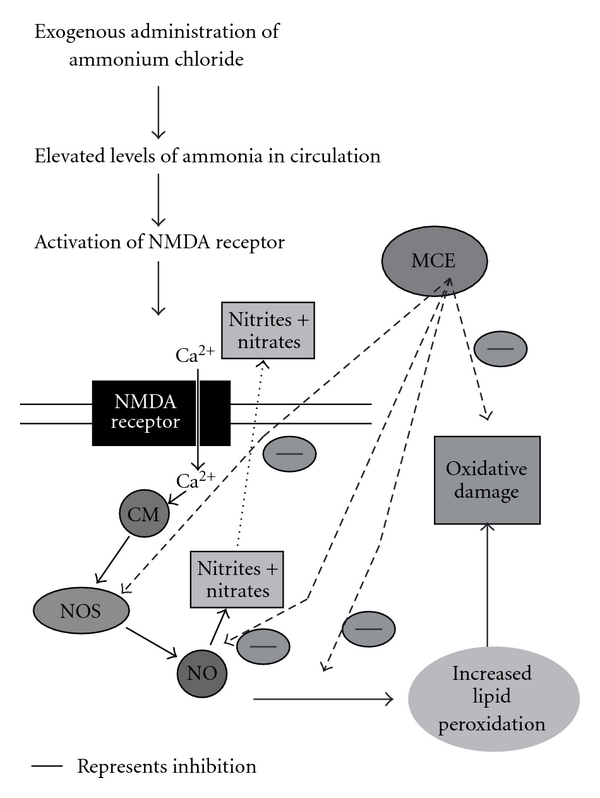
MCE attenuates the AC-induced oxidative damages. Thick line represents inhibition.

The elevated levels of circulatory liver markers and lipid peroxidation products in AC rats might be due to the liver damage caused by ammonia-induced free radical generation [37]. Reports have shown that excess ammonia intoxication leads to excessive activation of NMDA receptors leading to neuronal degeneration and death [38, 39]. The mechanisms by which excessive activation of NMDA receptors leads to neuronal degeneration and death are caused by the increased Ca^2+^ concentration in the postsynaptic neuron [40, 41]. Ca^2+^ binds to calmodulin and activates nitric oxide synthase (NOS), increasing the formation of nitric oxide (NO) that contributes to the neurotoxic process. Activation of NMDA receptors also leads to increased production of superoxide radical, which has also been observed under *in vivo* conditions [37, 42]. Superoxide and NO have the ability to generate hydroxyl radicals [43]. This leads to oxidative stress [1, 44], which results in increased levels of lipid peroxidation products and decreased levels of antioxidants in AC rats (Figure 1). Treatment with MCE in AC rats significantly increased the level of antioxidants with the depletion of lipid peroxidation products, which may be due to the inhibition of NOS and NO by MCE [45]. 

Under normal conditions, a dynamic equilibrium exists between the production of reactive oxygen species (ROS) and the antioxidant capacity of the cell [46]. Oxidative stress occurs when ROS levels exceed the antioxidant capacity of a cell. These ROS are highly toxic and react with lipids, proteins and nucleic acids, and lead to cell death via apoptosis or necrosis [47]. Peroxidation of lipids by the action of free radicals on unsaturated fatty acids has been implicated in the pathogenesis of various diseases [48]. Previous reports state that ammonium (chloride/acetate) salts induce ammonia toxicity partly via oxidative stress, which leads to lipid peroxidation and free-radical generation [37]. This could be the primary cause for the central nervous system malfunction associated with hyperammonemia. Infection, trauma or ingestion of large amounts of proteins is usually the precipitating factor causing hyperammonemia [49].

ROS may attack any type of molecules, but their main target appears to be polyunsaturated fatty acids (PUFAs), the precursors of lipid peroxide formation [50]. Free-radical damage to cellular components and decomposition of hydroperoxide formed from oxidative breakdown of PUFAs are important factors in the development of cellular toxicity and pathology caused by lipid peroxidation. It is now generally accepted that lipid peroxides play an important role in liver toxicity [51]. Treatment with MCE in hyperammonemic rats significantly decreased the levels of TBARS and HP in plasma. Earlier reports show that MCE could decrease TBARS during hypocholesterolemic conditions [52], due to its antilipidperoxidation and free-radical scavenging activity [53, 54], which coincides with the results of our experiments.

Serum AST, ALT and ALP are the most sensitive markers employed in the diagnosis of liver diseases. When the liver cell plasma membrane is damaged, numerous enzymes normally located in the cytosol are released into the blood stream [55], and their estimation in serum is a useful quantitative marker to indicate hepatocellular damage [56]. The increased activities of these serum markers observed in our study correspond to considerable liver damage induced in AC rats. Treatment with MCE significantly decreased the levels of AST, ALT and ALP, suggesting that they offer protection by preserving the structural integrity of the hepatocellular membrane against ammonium chloride, and our findings are in agreement with the previously published results [36, 57].

The ROS generation in tissues is efficiently scavenged by the enzymatic and nonenzymatic antioxidants. The decrease in the activities of antioxidant enzymes is in close relationship with the induction of lipid peroxidation [58]. Antioxidants play a major role in protecting biological systems from reactive oxygen-derived species and reflect the antioxidant capacity of the system [59]. The components of the defense system, which have evolved to reduce and contain the injury from free-radical attack, include several enzymes and a few free-radical scavenger molecules [60]. SOD plays an important role in protecting the cells from oxidative damage by converting superoxide radicals into hydrogen peroxide, which is further metabolized by CAT to molecular oxygen and water. In the present study, the decreased activity of SOD and CAT was observed in the liver and brain tissues of AC rats, and this decrease was antagonized when MCE was administered [61]. SOD is the first enzyme of the scavenger enzyme series to ameliorate the damage caused to cells by free radicals [62], while CAT is one of the several cellular antioxidant enzymes that provide a defense system for the scavenging of reactive oxygen metabolites. Possibly, the MCE used in this study might have accelerated the catabolism of H_2_O_2_ in hyperammonemic rats due to its superoxide-scavenging and powerful antioxidant activities [53], which corroborate our findings.

We observed decreased levels of GSH along with decreased activity of GPx in the liver and brain tissues of hyperammonemic rats. GSH is a major endogenous antioxidant, which counteracts free-radical-mediated damage [63]. It forms an important substrate for GPx and several other enzymes, which is involved in the free-radical scavenging [64]. In the tissues, GPx is a major enzymatic component for the disposal of peroxides, and a prolonged depression in the activity of this enzyme may lead to the intracellular peroxide accumulation. Decreased GSH levels might be due to increased utilization in protecting “SH"-containing proteins from lipid peroxides, and the unavailability of GSH may decrease the activities of GPx in AC rats. A previous report demonstrated that MCE could increase the level of GSH [61] and GPx [65] in diabetic rats, which coincides with our results. This may be due to the increased biosynthesis of GSH via activation of GSH synthase by MCE.

## 5. Conclusion

The biochemical findings of our present study indicate that MCE exerts protection to AC-induced hyperammonemic rats against oxidative stress. This could be due to the prevention or inhibition of lipid peroxidative system by its antioxidant, maintenance of cellular integrity and hepatoprotective effect. However, the exact mechanism is still unclear and further research on the effect of the constituents of this plant is needed.

## Figures and Tables

**Table 1:** Effect of MCE on changes in the blood ammonia and plasma urea, serum AST, ALT and ALP of normal and experimental rats.

Groups	Blood ammonia (*μ*mol L^−1^)	Urea (mg dl^−1^)	AST (IU l^−1^)	ALT (IU l^−1^)	ALP (IU l^−1^)
Normal	89.54 ± 4.47^a^	10.28 ± 2.99^a^	72.10 ± 6.26^a^	24.27 ± 2.08^a^	74.42 ± 6.14^a^
Normal + MCE (300 mg kg^−1^)	85.32 ± 8.94^a^	11.40 ± 0.77^a^	70.24 ± 6.28^a^	22.01 ± 1.78^a^	73.96 ± 4.50^a^
AC (100 mg kg^−1^)	327.15 ± 26.83^b^	22.93 ± 1.78^b^	118.41 ± 10.73^b^	60.34 ± 5.36^b^	141.72 ± 12.52^b^
MCE (300 mg kg^−1^) + AC	140.26 ± 17.88^c^	13.15 ± 0.89^c^	85.82 ± 8.74^c^	32.74 ± 2.68^c^	85.71 ± 7.15^c^

Each value is mean ± SD for 8 rats in each group. Values not sharing a common superscript (a, b and c) differ significantly at *P* < .05 (DMRT).

**Table 2:** Effect of MCE on the levels of TBARS and HP in plasma, liver and brain in normal and experimental rats.

Groups	Plasma TBARS (nM ml^−1^)	Plasma HP (values × 10^−5^ mM dL^−1^)	Liver TBARS (mM per 100 g wet tissue)	Liver HP (mM per 100 g wet tissue)	Brain TBARS (mM per 100 g wet tissue)	Brain HP (mM per 100 g wet tissue)
Normal	2.74 ± 0.17^a^	8.40 ± 0.11^a^	0.86 ± 0.07^a^	66.08 ± 5.21^a^	1.06 ± 0.08^a^	112.00 ± 8.50^a^
Normal + MCE (300 mg kg^−1^)	2.93 ± 0.17^a^	8.25 ± 0.54^a^	0.80 ± 0.06^a^	64.03 ± 5.12^a^	0.96 ± 0.07^a^	111.28 ± 8.48^a^
AC (100 mg kg^−1^)	4.56 ± 0.35^b^	13.16 ± 1.01^b^	2.16 ± 0.14^b^	97.86 ± 7.03^b^	1.97 ± 0.15^b^	135.24 ± 10.30^b^
MCE (300 mg kg^−1^) + AC	3.10 ± 0.17^c^	10.20 ± 0.89^c^	1.15 ± 0.11^c^	76.56 ± 5.81^c^	1.36 ± 0.11^c^	118.44 ± 2.76^c^

Each value is mean ± SD for 8 rats in each group. Values not sharing a common superscript (a, b and c) differ significantly at *P* < .05 (DMRT).

**Table 3:** Effect of MCE on the activities of SOD and CAT in the liver and brain of normal and experimental rats.

Groups	Liver SOD (U^a^ mg^−1^ protein)	Liver catalase (U^b^ mg^−1^ protein)	Brain SOD (U^a^ mg^−1^ protein)	Brain catalase (U^b^ mg^−1^ protein)
Normal	9.01 ± 0.58^a^	84.02 ± 6.30^a^	7.01 ± 0.47^a^	3.14 ± 0.23^a^
Normal + MCE (300 mg kg^−1^)	9.70 ± 0.73^a^	85.04 ± 6.45^a^	7.02 ± 0.48^a^	3.27 ± 0.19^a^
AC (100 mg kg^−1^)	3.78 ± 0.29^b^	40.98 ± 3.11^b^	5.28 ± 0.30^b^	0.87 ± 0.07^b^
MCE (300 mg kg^−1^) + AC	6.41 ± 0.38^c^	70.60 ± 5.37^c^	7.40 ± 0.35^c^	2.74 ± 0.21^c^

Each value is mean ± SD for 8 rats in each group. Values not sharing a common superscript (a, b and c) differ significantly at *P* < .05 (DMRT).

U^a^ − U^b^ is defined as the enzyme concentration required to inhibit the OD at 560 nm of chromogen production by 50% in 1 min. *P* < .05 (DMRT).

**Table 4:** Effect of MCE on the activities of GSH and the levels of GPx normal and experimental rats.

Groups	Liver GSH (mg per 100 g wet tissue)	Liver GPx (U^d^ mg^−1^ protein)	Brain GSH (mg per 100 g wet tissue)	Brain GPx (U^d^ mg^−1^ protein)
Normal	49.28 ± 3.02^a^	9.39 ± 0.70^a^	35.45 ± 2.69^a^	3.38 ± 0.25^a^
Normal + MCE (30 mg kg^−1^)	50.46 ± 4.31^a^	9.85 ± 0.74^a^	37.40 ± 2.76^a^	3.57 ± 0.27^a^
AC (100 mg kg^−1^)	24.86 ± 1.93^b^	5.06 ± 0.43^b^	20.94 ± 1.74^b^	1.19 ± 0.09^b^
MCE (30 mg kg^−1^) + AC	42.79 ± 3.20^c^	7.57 ± 0.72^c^	27.62 ± 2.16^c^	2.69 ± 0.20^c^

Each value is mean ± SD for 8 rats in each group. Values not sharing a common superscript (a, b and c) differ significantly at *P* < .05 (DMRT). U^d^: microgram of GSH consumed per minute.

## References

[1] Lena PJ, Subramanian P (2004). Effects of melatonin on the levels of antioxidants and lipid peroxidation products in rats treated with ammonium acetate. *Pharmazie*.

[2] Treem WR (1994). Inherited and acquired syndromes of hyperammonemia and encephalopathy in children. *Seminars in Liver Disease*.

[3] Monfort P, Felipo V (2005). Long-term potentiation in hippocampus involves sequential activation of soluble guanylate cyclase, cGMP-dependent protein kinase and cGMP-degrading phosphodiesterase alterations in hyperammonemia. *BMC Pharmacology*.

[4] Summar M, Tuchman M (2001). Proceedings of a consensus conference for the management of patients with urea cycle disorders. *Journal of Pediatrics*.

[5] Srinivasan K, Muruganandan S, Lal J, Chandra S, Tandan SK, Ravi Prakash V (2001). Evaluation of anti-inflammatory activity of *Pongamia pinnata* leaves in rats. *Journal of Ethnopharmacology*.

[6] Grubben GJH (1977). *Tropical Vegetable and Their Genetic Resources*.

[7] Ambasta SP (1986). *The Useful Plants of India*.

[8] Okabe H, Miyahara Y, Yamauchi T, Mirhara K, Kawasaki T (1980). Studies on the constituents of *Momordica charantia* L: I. Isolation and characterization of momordicosides A and B, glycosides of a pentahydroxycucurbitane triterpene. *Chemical & Pharmaceutical Bulletin*.

[9] Taylor L (2002). *Technical Data Report for Bitter Melon (*Momordica charantia*): Herbal Secrets of the Rainforest*.

[10] Jassim SAA, Naji MA *In vitro* evaluation of the antiviral activity of an extract of date palm (*Phoenix dactylifera* L.) pits on a Pseudomonas phage. *Evidence-Based Complementary and Alternative Medicine*.

[11] Chen Q, Chan LLY, Li ETS (2003). Bitter melon (*Momordica charantia*) reduces adiposity, lowers serum insulin and normalizes glucose tolerance in rats fed a high fat diet. *Journal of Nutrition*.

[12] Murakami T, Emoto A, Matsuda H, Yoshikawa M (2001). Structures of new cucurbitane-type triterpene glycosides, goyaglycosides-a, -b, -c, -d, -e, -f, -g, and -h, and new oleanane-type triterpene saponins, goyasaponins I, II, and III, from the fresh fruit of Japanese *Momordica charantia* L. *Chemical & Pharmaceutical Bulletin*.

[13] Parkash A, Ng TB, Tso WW (2002). Purification and characterization of charantin, a napin-like ribosome-inactivating peptide from bitter gourd (*Momordica charantia*) seeds. *Journal of Peptide Research*.

[14] Basch E, Gabardi S, Ulbricht C (2003). Bitter melon (*Momordica charantia*): a review of efficacy and safety. *American Journal of Health-System Pharmacy*.

[15] Muanda F, Kone D, Dicko A, Soulimani R, Younos C Phytochemical composition and antioxidant capacity of three Malian medicinal plant parts. *Evidence-Based Complementary and Alternative Medicine*.

[16] Shibib BA, Khan LA, Raman R (1993). Hypoglycemic activity of Coccinia Indica and *Momordica charantia* in diabetic rats: depression of the hepatic gluconeogenic enzymes glucose-6-phosphatase and fructose1, 6 biphosphatase and elevation of both liver and red cell shunt enzyme glucose-6-phosphate dehydrogenase. *Biochemical Journal*.

[17] Fernandes NPC, Lagishetty CV, Panda VS, Naik SR (2007). An experimental evaluation of the antidiabetic and antilipidemic properties of a standardized *Momordica charantia* fruit extract. *BMC Complementary and Alternative Medicine*.

[18] Subash S, Subramanian P (2008). Effect of morin on the levels of circulatory liver markers and redox status in experimental chronic hyperammonaemic rats. *Singapore Medical Journal*.

[19] Wolheim DF (1984). Preanalytical increase of ammonia in blood specimens from healthy subjects. *Clinical Chemistry*.

[20] Varley H, Gowenlock AH, Bell M (1998). *Practical Clinical Biochemistry, Vol. 1*.

[21] Reitman S, Frankel AS (1957). A colorimetric method for the determination of serum glutatmic oxaloacetic and glutamic pyruvic transaminases. *American Journal of Clinical Pathology*.

[22] King E, Armstrong AR (1934). Determination of serum and bile phosphatase activity. *Canadian Medical Association Journal*.

[23] Yagi K (1987). Lipid peroxides and human diseases. *Chemistry and Physics of Lipids*.

[24] Fraga CG, Leibovitz BF, Toppel AL (1988). Lipid peroxidation measured as TBARS in tissue slices. Characterization and comparison with homogenate and microsomes. *Free Radical Biology & Medicine*.

[25] Jiang Z-Y, Hunt JV, Wolff SP (1992). Ferrous ion oxidation in the presence of xylenol orange for detection of lipid hydroperoxide in low density lipoprotein. *Analytical Biochemistry*.

[26] Kakkar P, Das B, Viswanathan PN (1984). A modified spectrophotometric assay of superoxide dismutase. *Indian Journal of Biochemistry and Biophysics*.

[27] Sinha AK (1972). Colorimetric assay of catalase. *Analytical Biochemistry*.

[28] Ellman GL (1959). Tissue sulfhydryl groups. *Archives of Biochemistry and Biophysics*.

[29] Rotruck JT, Pope AL, Ganther HE, Swason AB (1973). Selenium: biochemical role as a component of glutathione peroxidase. *Science*.

[30] Lowry OH, Rosebrough MJ, Farr AL, Randall RJ (1951). Protein measurement with folin-phenol reagent. *The Journal of Biological Chemistry*.

[31] Kosenko E, Kaminsky Y, Lopata O (1998). Nitroarginine, an inhibitor of nitric oxide synthase, prevents changes in superoxide radical and antioxidant enzymes induced by ammonia intoxication. *Metabolic Brain Disease*.

[32] Nelson DL, Cox MM (2000). *Lehninger Principles of Biochemistry*.

[33] Essa MM, Subramanian P (2007). Hibiscus sabdariffa affects ammonium chloride-induced hyperammonemic rats. *Evidence-Based Complementary and Alternative Medicine*.

[34] Nakamura Y, Torikai K, Ohigashi H (2001). Toxic dose of a simple phenolic antioxidant, protocatechuic acid, attenuates the glutathione level in ICR mouse liver and kidney. *Journal of Agricultural and Food Chemistry*.

[35] Shirwaikar A, Malini S, Kumari SC (2003). Protective effect of *Pongamia pinnata* flowers against cisplatin and gentamicin induced nephrotoxicity in rats. *Indian Journal of Experimental Biology*.

[36] Abd El Sattar El Batran S, El-Gengaihi SE, El Shabrawy OA (2006). Some toxicological studies of *Momordica charantia* L. on albino rats in normal and alloxan diabetic rats. *Journal of Ethnopharmacology*.

[37] Essa MM, Subramanian P (2006). *Pongamia pinnata* modulates oxidant- antioxidant imbalance during hyperammonemic rats. *Fundamental & Clinical Pharmacology*.

[38] Beal MF (1992). Role of excitotoxicity in human neurological disease. *Current Opinion in Neurobiology*.

[39] Kosenko E, Kaminski Y, Lopata O, Muravyov N, Felipo V (1999). Blocking NMDA receptors prevents the oxidative stress induced by acute ammonia intoxication. *Free Radical Biology and Medicine*.

[40] Choi DW (1987). Ionic dependence of glutamate neurotoxicity. *Journal of Neuroscience*.

[41] Manev H, Favaron M, Guidotti A, Costa E (1989). Delayed increase of Ca^2+^ influx elicited by glutamate: role in neuronal death. *Molecular Pharmacology*.

[42] Hermenegildo C, Monfort P, Felipo V (2000). Activation of N-methyl-D-aspartate receptors in rat brain in vivo following acute ammonia intoxication: characterization by in vivo brain microdialysis. *Hepatology*.

[43] Hensley K, Tabatabaie T, Stewart CA, Pye Q, Floyd RA (1997). Nitric oxide and derived species as toxic agents in stroke, AIDS dementia, and chronic neurodegenerative disorders. *Chemical Research in Toxicology*.

[44] Norenberg MD, Rama Rao KV, Jayakumar AR (2004). Ammonia neurotoxicity and the mitochondrial permeability transition. *Journal of Bioenergetics and Biomembranes*.

[45] Lii C-K, Chen H-W, Yun W-T, Liu K-L (2009). Suppressive effects of wild bitter gourd (*Momordica charantia* Linn. var. *abbreviata ser*.) fruit extracts on inflammatory responses in RAW264.7 macrophages. *Journal of Ethnopharmacology*.

[46] Yu BP (1994). Cellular defenses against damage from reactive oxygen species. *Physiological Reviews*.

[47] Halliwell B, Gutteridge JMC (1989). *Free Radicals in Biology and Medicine*.

[48] Steinberg D, Parathasarathy S, Carew TE, Khow JC, Wiatum JL (1989). Modifications of low density lipoproteins that increase its atherogenicity. *The New England Journal of Medicine*.

[49] Bonnie R, Hu R (2008). Hyperammonaemia due to primary hyperparathyroidism-related renal tubular acidosis with incidental hypovitaminosis-D. *European Journal of Internal Medicine*.

[50] Gutteridge JMC (1982). Free-radical damage to lipids, amino acids, carbohydrates and nucleic acids determined by thiobarbituric acid reactivity. *International Journal of Biochemistry*.

[51] Rehman S, Mahdi AA, Hasan M (2003). Trace metal-induced lipid peroxidation in biological system. *SFRR-India Bulletin*.

[52] Ahmed I, Lakhani MS, Gillett M, John A, Raza H (2001). Hypotriglyceridemic and hypocholesterolemic effects of anti-diabetic *Momordica charantia* (karela) fruit extract in streptozotocin-induced diabetic rats. *Diabetes Research and Clinical Practice*.

[53] Wu S-J, Ng LT (2008). Antioxidant and free radical scavenging activities of wild bitter melon (*Momordica charantia* Linn. var. abbreviata Ser.) in Taiwan. *LWT—Food Science and Technology*.

[54] McCord JM, Fridovich I (1969). Superoxide dismutase. An enzymic function for erythrocuprein (hemocuprein). *Journal of Biological Chemistry*.

[55] Rajesh MG, Latha MS (2004). Preliminary evaluation of antihepatotoxic activity of Kamilari, a poly herbal formulation. *Journal of Ethnopharmacology*.

[56] Sallie R, Tredger JM, Williams R (1991). Drugs and the liver. *Biopharmaceutics and Drug Disposition*.

[57] Jayasooriya AP, Sakono M, Yukizaki C, Kawano M, Yamamoto K, Fukuda N (2000). Effects of *Momordica charantia* powder on serum glucose levels and various lipid parameters in rats fed with cholesterol-free and cholesterol-enriched diets. *Journal of Ethnopharmacology*.

[58] Jagetia GC, Rajanikant GK, Rao SK, Baliga MS (2003). Alteration in glutathione, glutathione peroxidase, superoxide dismutase, and lipid peroxidation by ascorbic acid in the skin of mice exposed to fractionated gamma radiation. *Clinica Chimica Acta*.

[59] Irshad M, Chaudhuri PS (2002). Oxidant-antioxidant system: role and significance in human body. *Indian Journal of Experimental Biology*.

[60] Vendemiale G, Grattagliano I, Altomare E (1999). An update on the role of free radicals and antioxidant defense in human disease. *International Journal of Clinical and Laboratory Research*.

[61] Chandra A, Mahdi AA, Ahmad S, Singh RK (2007). Indian herbs result in hypoglycemic responses in streptozotocin-induced diabetic rats. *Nutrition Research*.

[62] Slater TF (1984). Free-radical mechanisms in tissue injury. *Biochemical Journal*.

[63] Dewanjee S, Maiti A, Sahu R, Dua TK, Mandal V Effective control of type 2 diabetes through antioxidant defense by edible fruits of *Diospyros peregrine*. *Evidence-Based Complementary and Alternative Medicine*.

[64] Rahman I, MacNee W (1999). Lung glutathione and oxidative stress: implications in cigarette smoke-induced airway disease. *American Journal of Physiology*.

[65] Raza H, Ahmed I, John A, Sharma AK (2000). Modulation of xenobiotic metabolism and oxidative stress in chronic streptozotocin-induced diabetic rats fed with *Momordica charantia* fruit extract. *Journal of Biochemical and Molecular Toxicology*.

